# CD163+ immune cell infiltrates and presence of CD54+ microvessels are prognostic markers for patients with embryonal rhabdomyosarcoma

**DOI:** 10.1038/s41598-019-45551-y

**Published:** 2019-06-25

**Authors:** Jakob Nikolas Kather, Christian Hörner, Cleo-Aron Weis, Thiha Aung, Christian Vokuhl, Christel Weiss, Monika Scheer, Alexander Marx, Katja Simon-Keller

**Affiliations:** 1 Applied Tumor Immunity, German Cancer Research Center, Heidelberg, Germany; 2 Internal Medicine III, University Hospital RWTH Aachen, Aachen, Germany; 3 Institute of Pathology, University Medical Center Mannheim, Mannheim, Germany; 4 Center of Plastic-, Hand- and Reconstructive Surgery, University of Regensburg, Regensburg, Germany; 5 Institute of Pathology, Paidopathology, University Medical Center Kiel, Kiel, Germany; 6 Department of Medical Statistics and Biomathematics, University Medical Centre Mannheim, Mannheim, Germany; 7 Pediatrics 5 (Oncology, Hematology, Immunology), Olgahospital, Klinikum Stuttgart, Stuttgart, Germany

**Keywords:** Predictive markers, Cancer microenvironment

## Abstract

Rhabdomyosarcomas (RMS) are rare and often lethal diseases. It is assumed that the tumor microenvironment (TME) of RMS exerts an immunosuppressive function, but there is currently no systematic analysis of the immune cells infiltrating sarcoma tissue. Focusing on two common types of RMS (alveolar [RMA] and embryonal [RME]), we performed a comprehensive immunohistochemical analysis of tumor-infiltrating immune cells in the TME. We performed a qualitative estimation of infiltrating immune cells in the tumor microenvironment by an experienced pathologist as well as a quantitative digital pathology analysis. We found that (1) manual and automatic quantification of tumor-infiltrating immune cells were consistent; (2) RME tumors showed a higher degree of immune cell infiltration than RMA tumors but (3) the number of tumor infiltrating lymphocytes was low compared to other solid tumor types; (4) microvascular density correlated with immune cell infiltration and (5) CD163 positive macrophages as well as CD54 positive microvessels were more often detected in RME than in RMA and correlated with patient overall and event free survival. Our systematic analysis provides a comprehensive view of the immune landscape of RMS which needs to be taken into account for developing immunotherapies for this rare type of cancer.

## Introduction

Rhabdomyosarcoma (RMS) is a soft tissue malignancy that mainly affects children and young adults. Its main subtypes are alveolar RMS (RMA) and embryonal RMS (RME), both of which express skeletal muscle differentiation genes as defined by markers like myogenin (MYOG) and desmin but differ in terms of genetic and prognostic characteristics^1^. Whereas a specific genomic translocation between a PAX3 or PAX7 gene and FOXO1 (rarer translocation events also known) is the tumor initiating event of RMA, the prognostic more favorable RME is characterized by numerous genetic alterations^1^. The poor prognosis of RMA tumors is mainly traced back to early metastasis formation with five year survival rates below 30%^2^.

In general, immunotherapeutic options are considered as promising approaches to treat advanced cancer diseases. During the last decade it was shown that increasing the immune activity against tumor cells increases survival time in multiple solid tumor types^3,4^. In particular, blocking antibodies against immune checkpoint molecules such as PD-1, PDL-1 and CTLA-4 show clinically meaningful activity in a number of cancer types and have been clinically approved (for review see^5^). It is known that the best response rates are attained in tumors with high immunogenicity such as microsatellite-instable tumors^6^, melanoma^7^, non-small cell lung cancer^8,9^ and others. Other immunotherapy options include adoptive cell transfer of immune effector cells such as chimeric-antigen receptor T-cells (CAR-T) or tumor-infiltrating lymphocytes (TILs). A CAR-T treatment has been clinically approved for lymphatic tumors but not yet for solid tumors^10–13^. For RMS tumors we developed an adoptive immune therapy using chimeric T cells, that recognize the fetal acetylcholine receptor as tumor specific antigen^14^.

Various studies have shown that a successful cancer immunotherapy depends on a pre-existing anti-tumor immune response in the highly dynamic tumor microenvironment (TME)^15,16^. The TME is a complex system, consisting not only of tumor but also of endothelial cells, cancer associated fibroblasts (CAF), cytotoxic and immune suppressive immune cell infiltrates, antigen presenting cells and the extracellular matrix^17^ - a conglomerate that is generally called “tumor stroma”. The interactions between these constituents determine the clinical course of a solid tumor disease and the susceptibility to tumor immunotherapy^18,19^. Furthermore, enumerating the different cell types in histopathology yields powerful biomarkers that can be used to stratify patients and to predict treatment response^20^. Tumors with pronounced immune infiltrates are usually termed “hot”, while the absence of immune cells within the tumor tissue is referred to as a “cold” microenvironment. However, these quantitative categories alone do not adequately reflect the deeply complex immune infiltrate that is typically observed in human tumor tissue: besides infiltration with CD4+ T helper and CD8+ cytotoxic T cells, many tumors contain abundant immune suppressive cell types, like myeloid derived suppressor cells (MDSC), M2 macrophages and regulatory T cells (T_REG_ cells)^17^. The efficiency of immune therapeutic approaches based on cytotoxic immune cell infiltrates strongly depends on the composition of the TME. Especially, the interaction between tumor stroma together with tumor infiltrating lymphocytes strongly influences the outcome^19^. In summary, knowledge of the baseline (pre-therapy) immune infiltrate is helpful to predict for clinical course and treatment response and a detailed analysis of different cell types is necessary to derive clinical predictions^21^.

In the present study, we strived to deeply characterize the complex immune microenvironment of human RMS tumors. While the mutational landscape of sarcomas has been analyzed in large studies^22^, the immune landscape of sarcoma, especially RMA and RME is still understudied. Here, we employed serial stainings on a comparatively large patient cohort, using a large variety of specific markers for cytotoxic T cells, macrophages and immune suppressive regulatory T cells. To the best of our knowledge, this is the first study that systematically addresses the immune contexture of RMA and RME: In particular, the stainings were independently analyzed by a previously validated computer based approach^23^ and an experienced pathologist, thereby combining the assessment of the pathologist with the quantitative analysis of the computer based approach.

## Results

### Implementation of a computer-based image analysis

**Figure 1:**
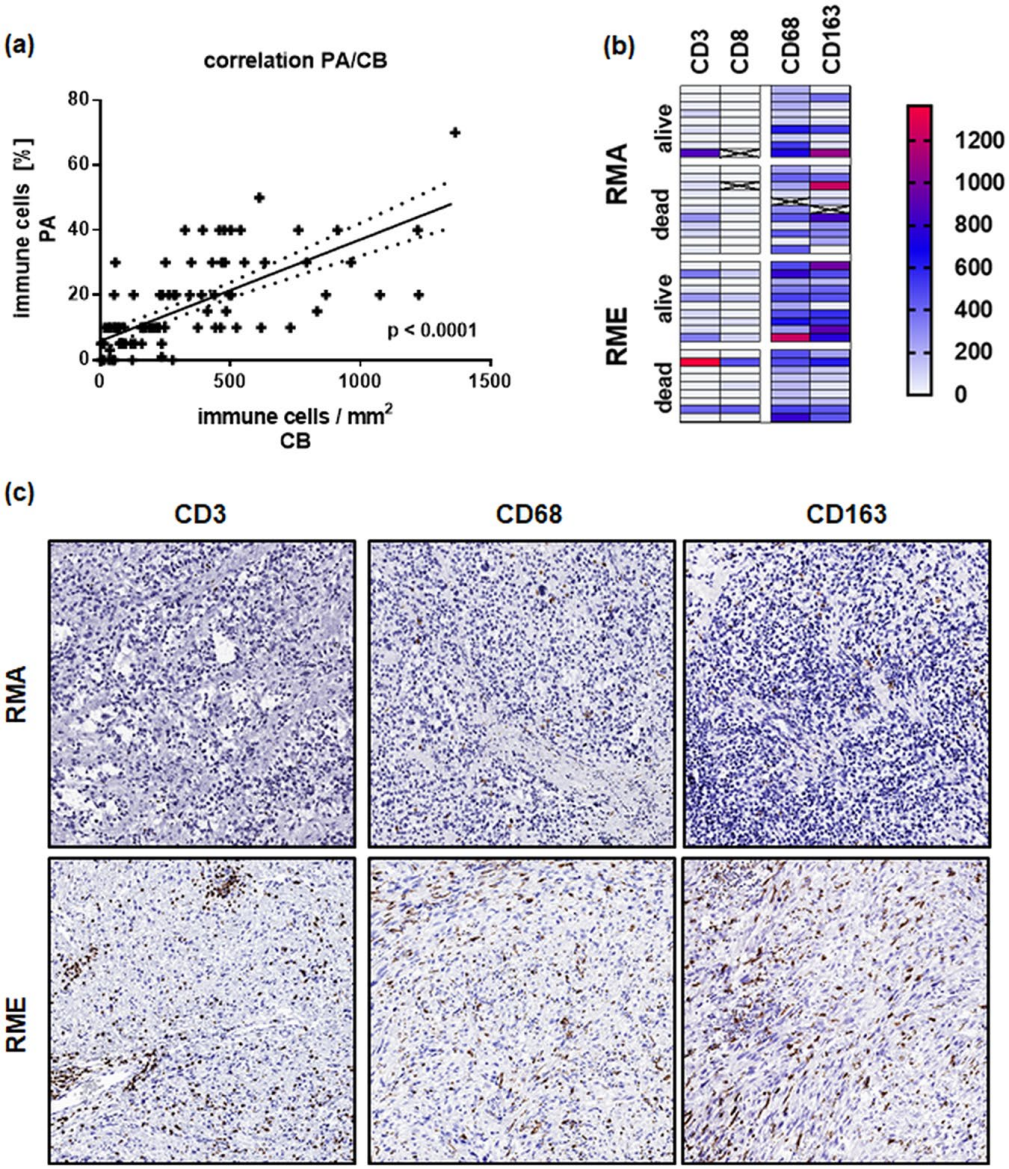
(**a**) Respresents the correlation between the estimated immune cell amount done by an experienced pathologist (PA; y-axis; immune cell infiltrates in percent) and the computer based quantification (CB; x-axis; immune cell infiltrates/mm^2^). (**b**) Distribution of immune infiltrating cells in RMA and RME tumors presented as heat map. (**c**) Shows representative stainings for CD3, CD68 and CD163 in RMA and RME tumors; magnification 100x. RMA – alveolar Rhabdomyosarcoma; RME – embryonal Rhabdomyosarcoma.

We systematically quantified tumor-infiltrating immune cells in RMS independently by a manual and an automatic approach. The immune infiltrates were semiquantitatively assessed by an experienced pathologist (A.M.) and compared to the computer-based cell densities of CD3, CD68 and CD168 cells per mm^2^, which gave comparable and highly correlated results (Fig. 1a). Hereinafter, the quantified cell numbers based on the computer approach were used for data analysis, if not otherwise indicated. Intra-tumoral and septal regions were not subdived by the computer based approach as it was done by the pathologist. However, tumor regions were defined and separated from peripheral regions, whenever normal tissue was visible in addition to the tumor. Figure 1b, c represented the distribution of the analyzed cell counts in RMA and RME tumors.

### Infiltration of RMS tumors with CD3^+^ immune cells

**Figure 2:**
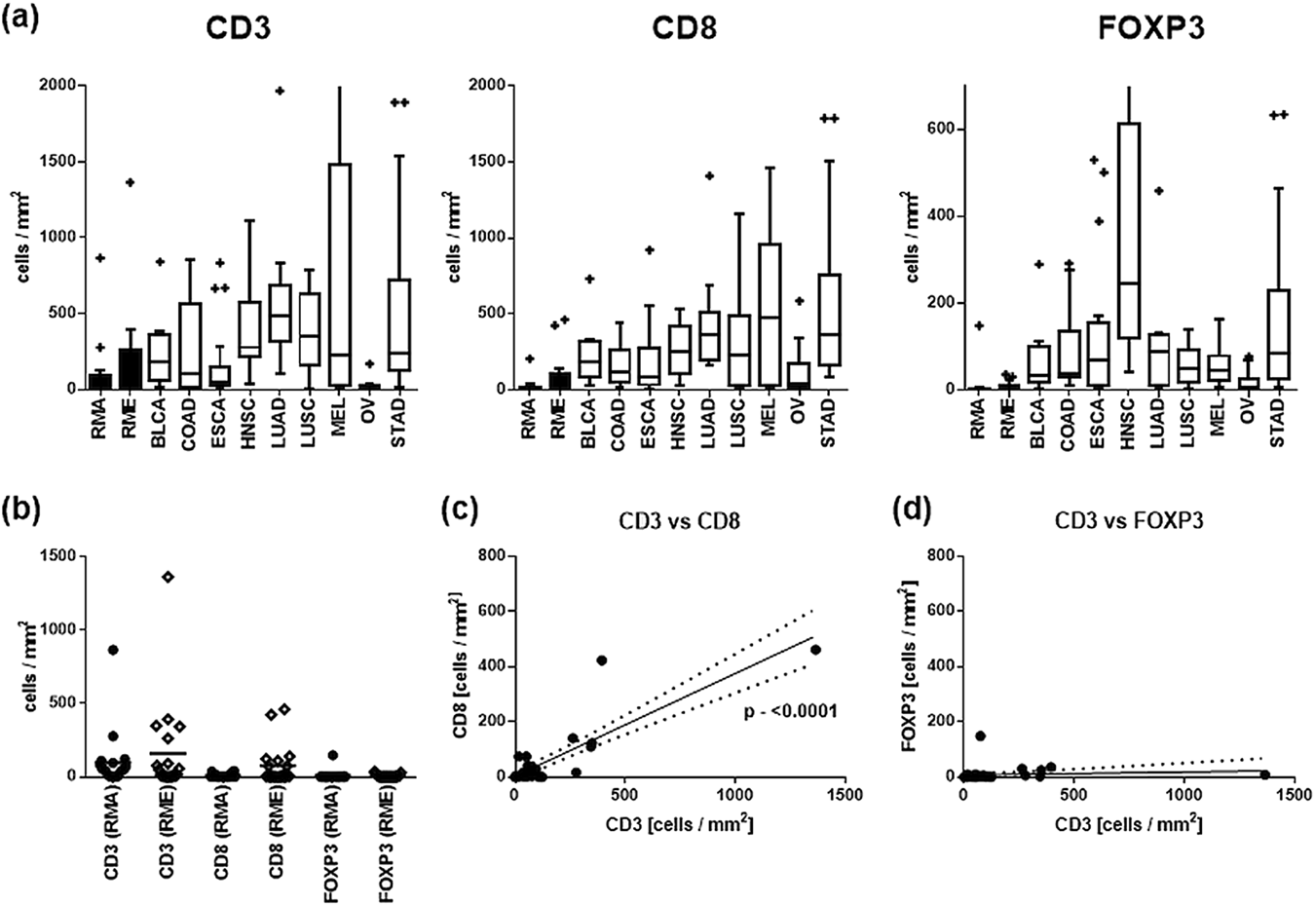
(**a**) Box plots showing the infiltrating immune cells in RME and RMA tumors compared to other tumor entities. The boxes extend from the 25^th^ to 75^th^ percentile, the horizontal line in the box represents the median, for the location of the wiskers the turkey method was choosen. (**b**) Shows an overall comparison of CD3 infiltrating cells with CD8 and FOXP3 in RMA and RME, single samples and the corresponding mean values are shown. (**c**) Shows the correlation between CD3 and CD8 infiltrating cells in RMS as well as (**d**) CD3 and FOXP3 infiltrating cells. RMA – alveolar Rhabdomyosarcoma; RME – embryonal Rhabdomyosarcoma; BLCA – bladder carcinoma; COAD – colorectal adenocarcinoma; ESCA - esophageal squamous carcinoma; HNSC - head and neck squamous cell carcinoma; LUAD - lung adenocarcinoma; LUSC - lung squamous cell carcinoma; MEL – melanoma; OV - ovarian cancer; STAD - gastric cancer.

Overall, lymphocyte densities were low compared with the recently published dataset^23^ (Figs 1b and 2a) and highly heterogeneous within a given examined tumor sample, with immune cell hotspots in some areas and no detectable lymphocytes in other areas of the tumor. In general, we found fewer CD3 positive T lymphocytes in RMA than in RME (Fig. 2b). However, the differences between RME and RMA were only significant (p < 0.05) when the manually determined immune cell percentages were compared between RMA and RME. This is probably because the computer based approach also rate very low numbers of immune cell infiltrates, which the pathologist considered as negative or less than one percent relevant immune cells/area. By excluding very low numbers (less than 20 cells/mm2) from the calculation, a better separation between RME and RMA tumors is shown again (Supplemental Fig. S1). Subsequent analyses, however, were performed with the computer-based variant and very low numbers of infiltrating cells were also taken into account, if not otherwise indicated.

The low number of tumor-infiltrating CD3+ T cells implied low numbers of cytotoxic T cells in RMA and RME as compared to other tumor types (Fig. 2a). Indeed, CD8 T cell numbers were generally sparse in RMS. When comparing RMA and RME, we found more CD8 T cells in RME tumors (Fig. 2b and Supplemental Fig. S1). As expected, a strong correlation between CD3 and CD8 infiltrating immune cells is detectable (Fig. 2c and Supplemental Table S1). In addition, almost no infiltration of RMS tumors with CD3^+^/FOXP3^+^ regulatory T cells (T_REG_) were detectable (Fig. 2a, b and d).

### Infiltration of RMS with macrophages

**Figure 3:**
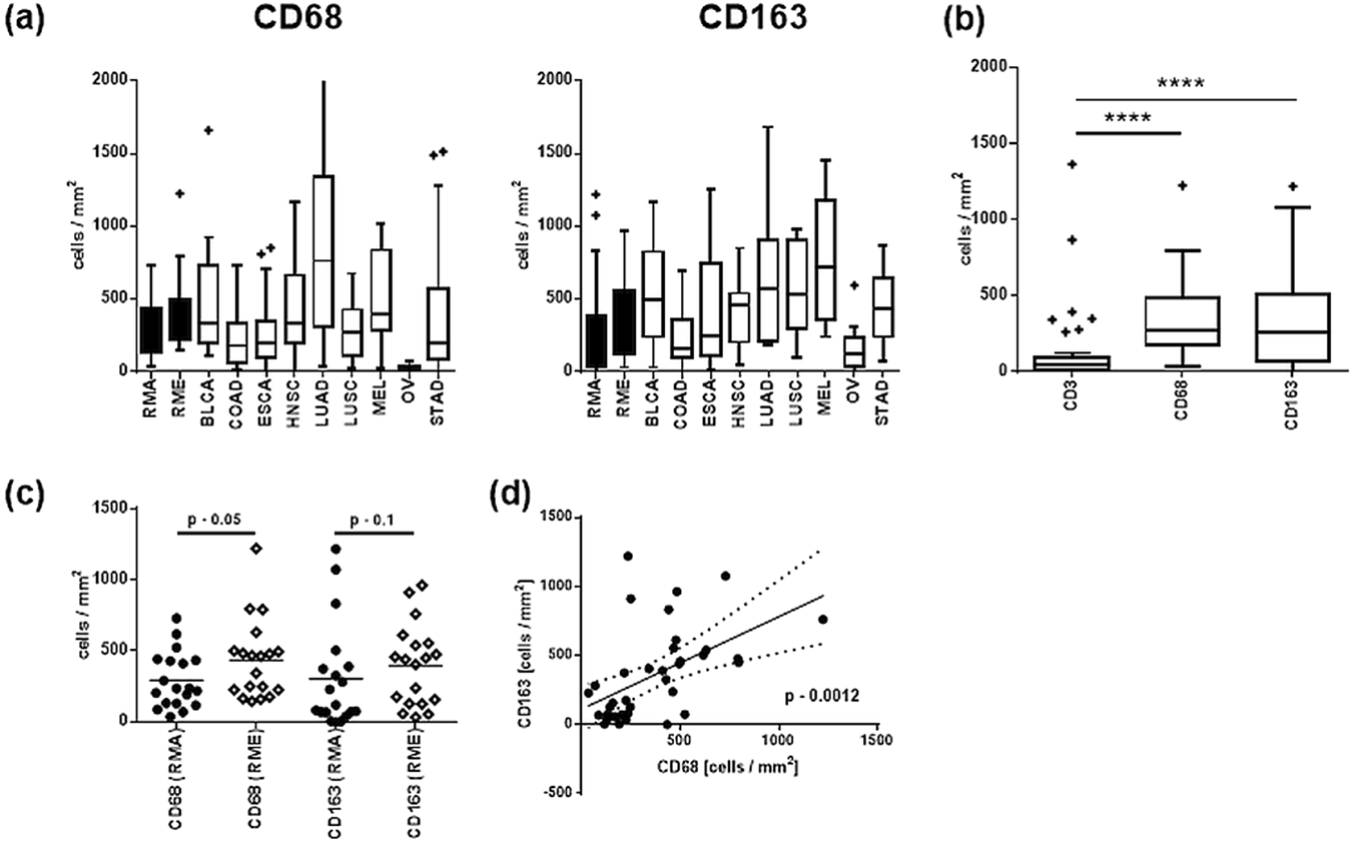
(**a**) Box plots showing the infiltrating immune cells in RME and RMA tumors compared to other tumor entities. (**b**) Box plots of the CD3, CD68 and CD163 infiltrating cells in RMS. The boxes extend from the 25^th^ to 75^th^ percentile, the horizontal line in the box represents the median, for the location of the wiskers the turkey method was choosen. (**c**) Shows a comparision of CD68, CD163 infiltrating cells in RMA and RME tumors; single samples and the corresponding mean values are shown. (**d**) Shows the correlation between CD68 and CD163 cells in RMS tumors. RMA – alveolar Rhabdomyosarcoma; RME – embryonal Rhabdomyosarcoma; BLCA – bladder carcinoma; COAD – colorectal adenocarcinoma; ESCA - esophageal squamous carcinoma; HNSC - head and neck squamous cell carcinoma; LUAD - lung adenocarcinoma; LUSC - lung squamous cell carcinoma; MEL – melanoma; OV - ovarian cancer; STAD - gastric cancer; *p < 0.05, ** 0.05 > p > 0.01, *** 0.01 > p > 0.005, ****p < 0.005.

Analysis of the macrophage markers CD68 and CD163 revealed that intra-tumoral macrophages were overall more abundant than lymphocytes in the whole RMS group (on average 130 CD3^+^ cells/mm^2^ vs 360 CD68^+^ cells/mm^2^ and 350 CD163^+^ cells/mm^2^) as well as the subsets (RMA: 100: 290: 300; RME: 160: 430: 400) (Fig. 3a,b). As was observed for T cells, intra-tumoral densities of macrophages were more numerous (in terms of CD68 and CD163) in RME than RMA (Fig. 3c). The number of CD68 positive cells correlates with the number of CD163 positive cells (p = 0.04) (Fig. 3d). This was also the case for the RMS subtypes (Supplemental Table S1). In addition, an association of CD3 and CD68 or CD163 positive macrophages was detectable. This association was more pronounced in RMA tumors as shown in Table S1.

### B-lymphocytes and expression of PD1 and PD-L1

**Figure 4:**
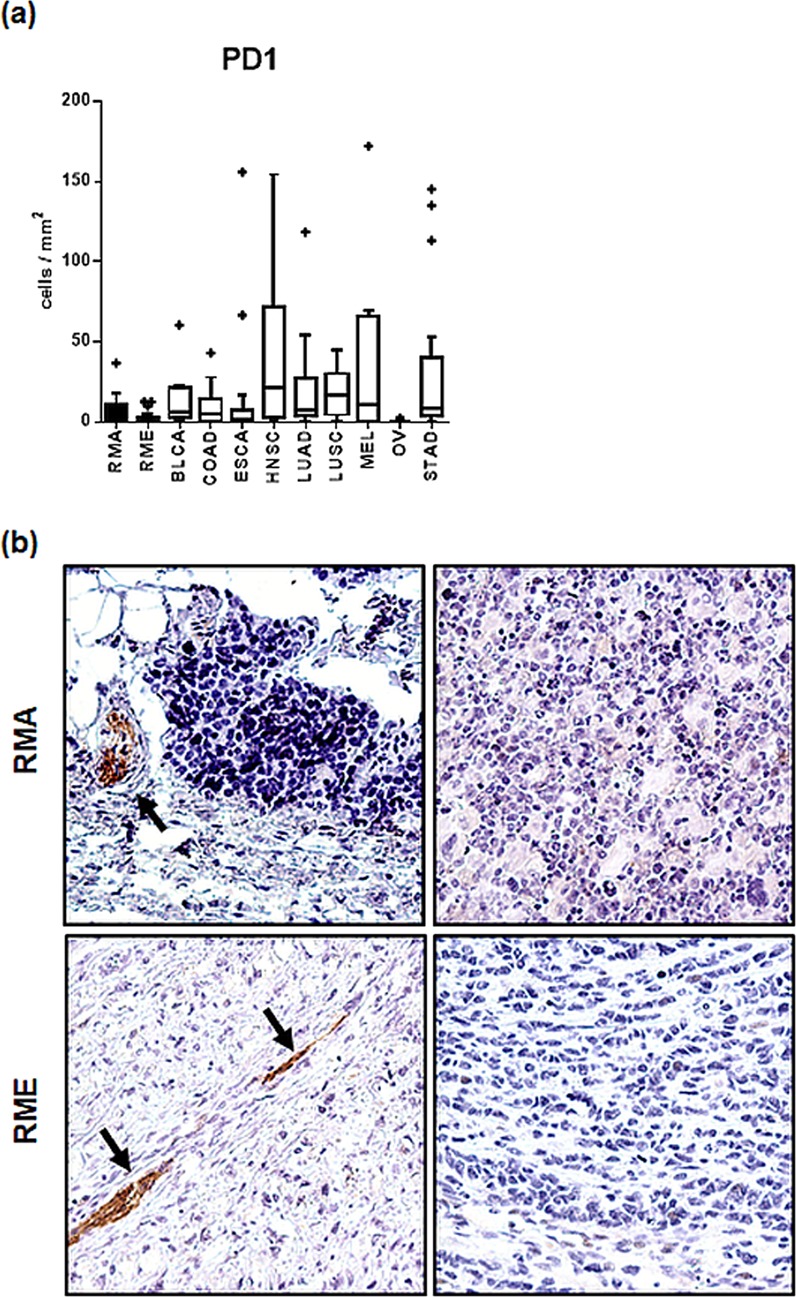
(**a**) Box plots showing PD-1 positive cells in the tumor microenvironment of RME and RMA tumors compared to other tumor entities. The boxes extend from the 25^th^ to 75^th^ percentile, the horizontal line in the box represents the median, for the location of the wiskers the turkey method was choosen. In terms of PD-L1 expression (**b**) shows lack of PD-L1 expression in four representative RMS, two RME and two RMA; tumor associated nerves were positive^61^ and served as internal control (arrows); maginification – 200x. RMA – alveolar Rhabdomyosarcoma; RME – embryonal Rhabdomyosarcoma; BLCA – bladder carcinoma; COAD – colorectal adenocarcinoma; ESCA - esophageal squamous carcinoma; HNSC - head and neck squamous cell carcinoma; LUAD - lung adenocarcinoma; LUSC - lung squamous cell carcinoma; MEL – melanoma; OV - ovarian cancer; STAD - gastric cancer.

CD20 positive B-lymphocytes were but exceptionally observed and occurred only in follicles in the peritumorous region, but not in the tumor itself (Supplemental Fig. S2). The immune suppressive surface molecule PD1 was not expressed on tumor cells and only on exceedingly rare intratumoral mononuclear cells in a small minority of RMA and RME (Fig. 4a), while PD-L1 expression was consistently negative on tumor cells and tumor infiltrating immune cells in all RMS cases investigated (Fig. 4b). Of note, in the minority of cases with peritumoral lymphoid follicles, some cells inside these follicles stained for PD1 (supposedly T cells) and PD-L1 (supposedly dendritic cells) (Supplemental Fig. S2).

**Figure 5:**
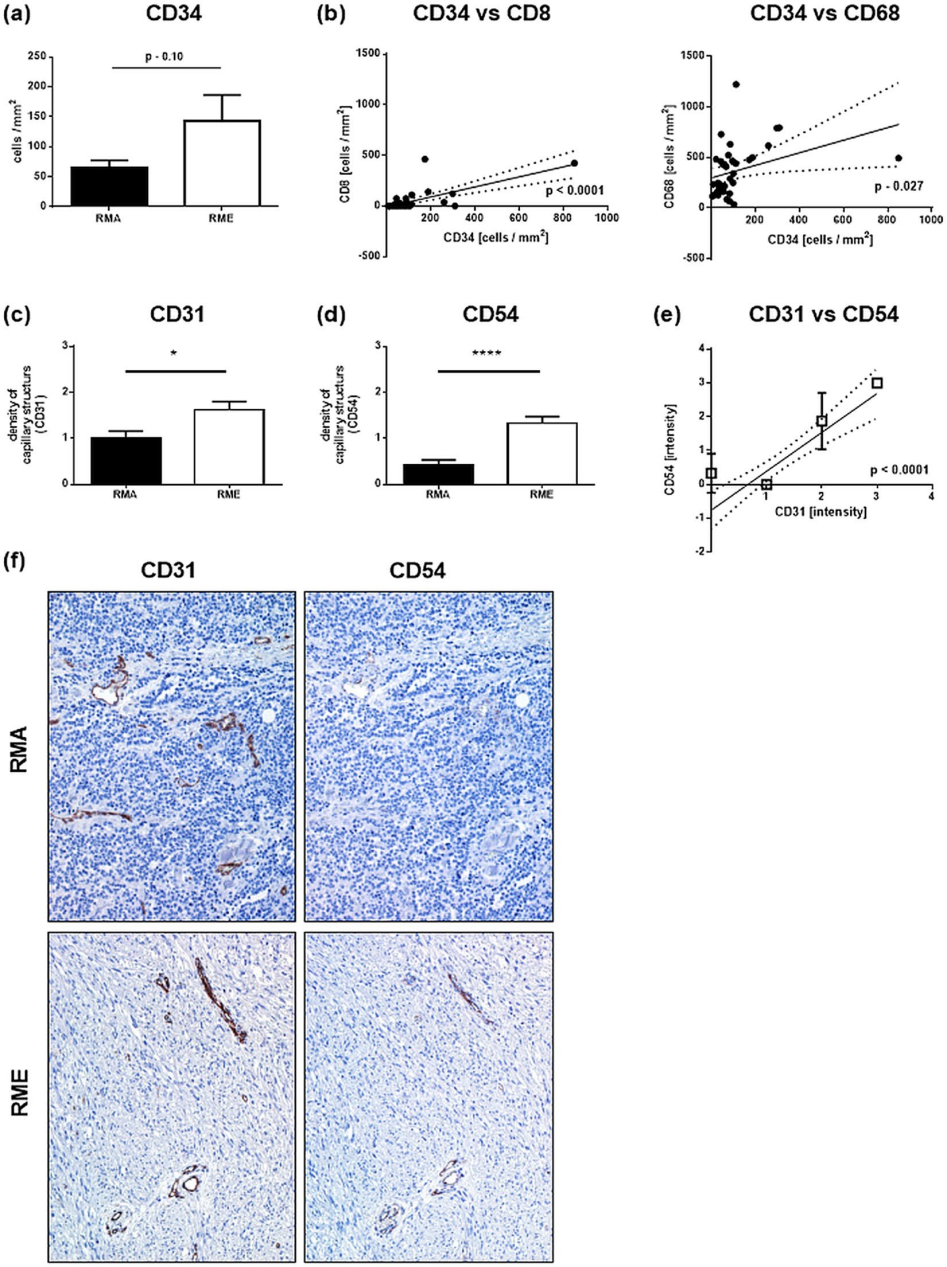
(**a**) Comparison of CD34 positive microvessels in RMS subtypes, RMA and RME. (**b**) Shows the correlation between CD34 positive microvessels and CD8 (left panel) or CD68 (right panel) infiltrating cells in RMS. (**c**) Comparison of CD31 positive microvessels and (**d**) CD54 positive microvessels in RMA and RME tumors. (**e**) Shows a correlation analysis of CD31 and CD54 positive microvessels in RMS. In (**f**) a sequential staining of CD31 and CD54 was performed for RMA and RME tumors. For each subtype a representative staining is shown; magnification – 100x. (**a**), (**c**) and (**d**) display the mean values with the respective standard error of the mean; RMA – alveolar Rhabdomyosarcoma; RME – embryonal Rhabdomyosarcoma, *p < 0.05, ** 0.05 > p > 0.01, *** 0.01 > p > 0.005, ****p < 0.005.

To examine the microvasculature as a precondition for the infiltration of tumors with immune cells, sections were stained with the endothelial markers CD31 and CD34. Again, CD34 postive cells were slightly more numerous in RME than RMA (Fig. 5a). Moreover, correlation analysis revealed an assocation of CD8 and CD68 with CD34 positive cells (p < 0.0001 and p = 0.027) (Fig. 5b) and a trend for association of CD3 with CD34 positive cells (not shown). For CD8 this association was also observed in the subtypes (Supplemental Table S2).

Quantitative assessment of microvessel density based on automatic analysis of CD34 stained sections (Fig. 5a and Supplemental Fig. S3, respectively) revealing higher amounts of microvessels in RME compared to RMA tumors (p = 0.01 for CD34, p = 0.026 for CD31). Being blinded to the morphometric data, the pathologist (A.M.) independently confirmed this finding using semiquantitative estimation of vessel density in CD31 and CD34 stainings (Fig. 5c and Supplemental Fig. S3). In addition, for RME tumors a significant higher number of CD54 positive vessles were detectable (Fig. 5d), which was positively correlated with CD31 positive capillaries (Fig. 5e). Indeed, sequentiell staining of RME and RMA tumors showed more CD31/CD54 postive capillaries in RME tumors than RMA tumors (Fig. 5f). Furthermore, independent analysis of tumors with high density of CD54 positive microvessels (grading 2; CD54^HIGH^) and tumors with a low densitiy of CD54 positive capilary structures (grading 0 and 1; CD54^LOW^) showed that there is a strong association between the grade of CD54 positivity of capillary sructures and the number of infiltrating CD3, CD8, CD68 and CD163 immune cells (Fig. 5f and Table 1). The subsequent analyses of the association between the extent of endothelial CD54 expression and survival (see below) were only meaningful for RME tumors, since RMA tumors consistently showed a CD54^LOW^ endothelial phenotype (i.e. grade 0 or 1 microvessel density and CD54^LOW^ expression levels).

**Table 1:** Association of CD54 intensity with the number of CD3/CD8/CD68 and CD163 immune cells; p-values are given (Kruskal-Wallis-Test).

	RMS all	RME
CD54/CD3 association	0.019	0.021
CD54/CD8 association	0.006	0.021
CD54/CD68 association	0.004	0.027
CD54/CD163 association	0.008	0.063

### Survival

We analyzed a cohort of 39 tumor patients of which 11 RMA and 9 RME patients died from cancer during the follow-up period. Analysis of the event free survival (EFS) for both subgroups revealed a better EFS for RME patients and for patients with a more favorable localization, but both without reaching significance (Supplemental Table S4 and Fig. S4).

**Figure 6:**
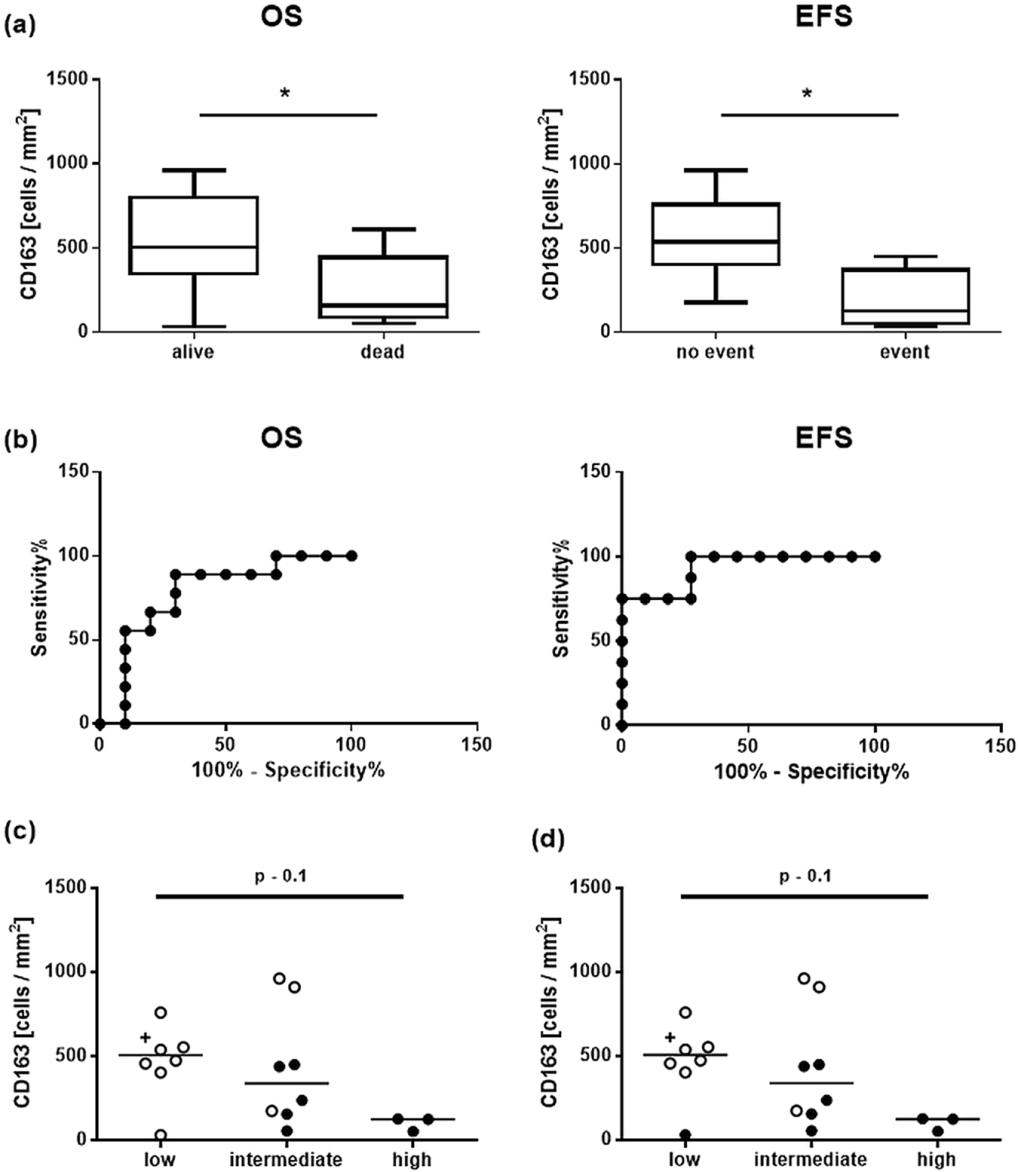
(**a**) Overall (OS) and event free (EFS) survival of patients with CD163+ immune cell infiltrates in RMS tumors. (**b**) Representative ROC curves of OS and EFS from 6a. (**c**) + (**d**) Show the numbers of CD163 positive cells in the tumor microenvironment of low, intermediate and high risk RME patients. Due to a strongly skrewed distribution the median is shown. One patient (shown as cross) in the group “low” risk showed an unusually high density of immune cells and was not considered in the present statistical analysis (see also Supplemental Fig. S5), but shown in the illustration for reasons of transparency. Open circles represent patients who, (**c**) did not die or (d) had no detectable event during the observation period; OS – overall survival; EFS – event free survival, *p < 0.05, ** 0.05 > p > 0.01, *** 0.01 > p > 0.005, ****p < 0.005.

For the whole group of RMS (n = 39), neighter the amount CD3 nor CD8 or CD68 infiltrating cells was associated with OS or EFS. Nevertheless, a logistic regression analysis for the RMA and RME subtypes revealed an influence of CD163 on the mortality (dead yes or no) of RME tumor patients (p = 0.04) with an ODDS ratio of 0.634 and the event free survival time (p = 0.004) with an ODDS ratio of 0.497 (Fig. 6a). This means, the probability for an event was significant lower in RME patients with a higher number of CD163 infiltrating immune cells. Indeed a subsequent ROC analysis revealed CD163 as a good predictive marker with a cut-off of 237 infiltrating CD163 cells per mm^2^ (with a sensitivity of 0.7 and a specificity of 0.88) (Fig. 6b).

Separation of the available RME set in patients with low, intermediate or high risk tumors revealed more numerous tumor infiltrating CD3 and CD8 positive cells in patients with low risk tumors (Supplemental Fig. S5) compared to intermediate and high risk tumors. For CD68 and CD163 no difference was detectable between low and intermediate risk tumors, but both groups showed a denser infiltration with CD68 and CD163 positive cells compared to high risk tumors (Fig. 6c and Supplemental Fig. S5). Further differentiation of the data set into patients who died of their tumors (black dots in Fig. 6c and Supplemental Fig. S5) or patients who showed tumor progression and/or metastasis (black dots in Fig. 6d and Supplemental Fig. S5) during the observation period, revealed two patients with intermediated risk tumors, but high numbers of CD163 positive cells in the tumor microenvironment (Fig. 6c,d). Both patients showed overall and event free survival of 2.8 and 7.8 years, respectively, compared to the other patients in the intermediate risk group with event free survival of less than 1.5 years. Vice versa, one patient in the low risk group showed a particularly low number of immune-infiltrating CD163 positive cells and tumor progression during the observation period (EFS 1.5 year) (Fig. 6c, d).

**Figure 7:**
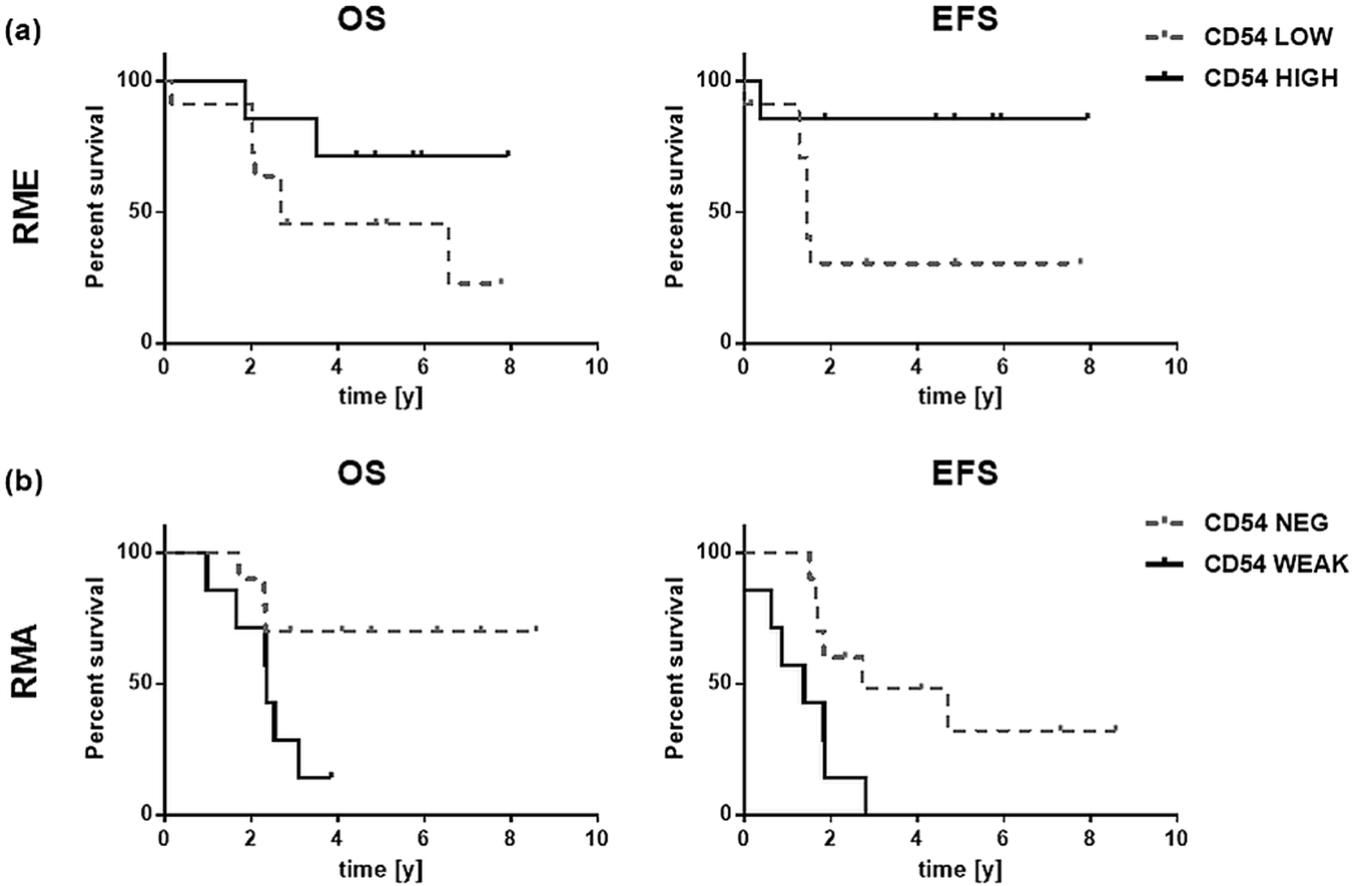
(**a**) Shows OS and EFS of RME patients with CD54 high and low phenotypes. (**b**) Shows OS and EFS of RMA patients without CD54 positive microvessels and low numbers of CD54 positive vessels; OS – overall survival; EFS – event free survival; y – years.

Since a higher abundance of CD54 positive microvessels was associated with a higher number of immune cells, we tested the survival of RMS patients with CD54^HIGH^ and CD54^LOW^ phenotypes. Again, in the cohort of all RMA and RME patients either CD54 phenotype was not associated with a better OS or EFS. However, the separation into RME and RMA subtypes showed a tendency to better OS (p = 0.20) and EFS (p = 0.05) survival of patients with CD54^HIGH^ RME (Fig. 7a, Supplemental Table S3). Since RMA tumors consistently showed a CD54^LOW^ phenotype they were analyzed by dividing capillary density into 0 (no CD54 capillaries detectable) and 1 (only few CD54 positive capillaries detectable). Contrary to what we had expected from the findings in RME, this revealed a better OS (p = 0.05) and EFS (p = 0.02) of patients with RMA tumors that lacked CD54-positive endothelial cells (grade 0) (Fig. 7b, Supplemental Table S3).

## Discussion

Immune therapies are the new hopes for otherwise refractory cancers, and better knowledge of the tumor microenvironment is considered key to predict treatment outcome^24^. Therefore, we performed a comprehensive quantitative digital pathology-based analysis of various intratumoral immune cells, PD1 and PD-L1 expression and microvascular features in RMA and RME with reference to the clinical outcome, from which we gained the following new findings: (1) RMA exhibited a low number of intratumoral CD3+ and CD8+ T cells, while the abundance of these cells was slightly higher among RME cases; (2) FOXP3 positive regulatory T cells (and B cells) were generally absent from RMA and RME; (3) CD54+ microvessels were significantly more frequent in RME than RMA and their presence was associated with higher numbers of intratumoral immune cells; (4) high numbers of intratumoral CD163+ macrophages and CD54+ microvessels were more common in RME and associated with improved survival in RME. Furthermore, we confirm the overall low abundance or even absence of CD8+ T cells in RMS as compared to other pediatric sarcomas^25–27^, and can now add that intratumorous lymphoid cells are also less abundant in RMS than in a broad spectrum of common cancers of adulthood^23^.

The present systematic analysis of RMS and comparison with the 10 most prevalent non-sarcoma neoplams that are currently the focus of immunotherapies^28^, revealed an overall paucity of intratumoral CD3+ and CD8+ lymphoid infiltrates in RMS. However, different neoplasms (carcinoma, melanoma, sarcoma) differ in terms of immune cell activation and infiltration^29^. Even studies of soft tissue sarcomas revealed that the immune cell microenvironment differentally affected outcome and prognosis in different sarcoma subtypes^30–32^. A comprehensive analysis of 249 soft tissue sarcoma (including 16 RMS tumors) showed that – across a broad spectrum of different ifiltrating immune cells analyzed - only an infiltration with CD20 positive B cells was an independent favorable prognostic marker^31^, i.e. a lymphocyte subset that was completely missing in our tumor samples. This shows that immunobiological findings obtained in a tumor entity should not be generalized without further ado, and special caution is to be taken when comparing childhood and adult cancers.

Although RMS together with Ewing sarcoma, neuroblastoma, glioblastoma and medulloblastoma frequently show lymphocytic infiltrates^33^, the total number of infiltrating immune cells in these tumor subtypes, as far as comparable quantitative studies are available, is low^27,34–36^ compared to the adult tumor entities analyzed in our study (Supplemental Fig. S6). Hence, it is tempting to speculate that childhood cancers in general and RMS in particular, are largely ignored by the adaptive immune system because their total mutational burden (TMB) on average is among the lowest of all human cancers^37–39^, while solid tumors that commonly elicit a vigorous local inflammation (e.g. melanomas and lung cancers) typically show high TMB^21,22,40–43^. This hypothesis is also compatible with the observed higher number of immune cells in RME that show more genetic instability than RMA^37^. Futhermore, the virtual absence of FOXP3+ cells in the microenvironment of both RMA and RME; the lacking expression of the immunoregulatory PD-L1 and PD1 in any of our RMS cases (contrary to some mouse models^44^) and the preferential reduction of CD8+ cells over intratumoral macrophages in RMS as compared to a broad spectrum of other cancers (Figs 2 and 3)^23^, are all compatible with a scenario of “immunological ignorance” by the adaptive immune system rather than suppression of a specific cytotoxic immune response or one of the typical strategies of tumors to escape from a cytotoxic attack, namely PD-L1 upregulation^43^.

Therefore, future biomarker studies may address the question whether the observed heterogeneity of inflammatory infiltrates in RMS (particularly in RME), reflects the heterogeneity of TMB. Such studies should preferably include cases of an apparently small RMS subset (that we failed to identify in our cohort) with expression of PD-L1 on RMS tumor cells and excellent clinical outcome^45^.

An additional reason for the paucity of intratumoral lymphocytes in RMS could be the paucity of activated, CD54+ microvessels that we observed in a subset of RME and almost all RMA. The positive correlation between intratumoral lymphocyte numbers and endothelial CD54 expression in our cohort of RMS is in line with studies showing improved, CD54-dependent recruitment of leukocytes to the microenvironment of various non-rhabdomyomatous tumors^24,46^. Although it appears likely that the strong focal endothelial expression of CD54 accompanied by focal lymphoid cell infiltrates in a subset of RMS are interrelated by a local immune reaction, it is unknown whether the deficient or low (grade 0–1) endothelial expression of CD54 in many RMS (and most RMA) reflects lack of endothelial activation or suppression of CD54 expression (“endothelial anergy”) that is often encountered in tumor microenvironments (e.g. through proangiogenic factors that are generated due to intratumoral hypoxia)^47^.

Like lymphoid cells, CD68+ and CD163+ macrophages were less abundant in RMS than in the most common cancers of adulthood (Figs 2 and 3). However, macrophage numbers in RMS were less strikingly reduced than the numbers of CD3+ cells, suggesting that innate immune mechanisms via the recruitment of myeloid cells to the TME of RMS might play a greater immunological role than adaptive immunity in RMS - with mechanisms of monocyte homing to the TME (e.g. through chemokines and growth factors^48^) awaiting elucidation. The positive correlation between the number of macrophage and CD54+ microvessels in RMS might mirror the well known production of angiogenic factors by tumor associated macrophages^49^. By contrast, the observation that higher numbers of CD163 positive macrophages and CD54+ microvessels were *both* associated with better survival of RME patients was unexpected: In almost all cancers high numbers of macrophages herald a poor prognosis that is thought to reflect the role of macrophages as suppressors of antitumor immunity and promoters of invasion and metastasis^48^. The opposite association encountered here in RME is, however, not without precedent: Cunha *et al*. found high numbers of macrophages in thyroid cancers linked with favorable outcome, making the authors propose two possible explanations for their exceptional finding: First, macrophages might activate rather than suppress the numerous CD8+ T cells that consistently accompany macrophages in thyroid carcinomas; second, a direct antitumor phagocytic effect of macrophages could be operative^50^. As to the first option, there is evidence of macrophage functional placity, i.e. their potential to switch from an immunoregulatory to an immunostimulatory function due to environmental cues or pharmacological intervention^48,51,52^. However, in light of the paucity of intratumoral T cells compared to the relative abundance of macrophages in the TME of RMS, the second mechanism may also be operative considering the high susceptible of RMS to macrophage-mediated cytotoxicity *in vitro*^53^. This is supportet by our finding, that especially low risk and a group of patients with intermediate risk tumors showed higher infiltration with CD163 positive macrophages.

In any case, more investigations of the TME of RMS are necessary to learn whether the better prognosis of CD54+ microvessel-rich compared to microvessel-poor RME is due to better recruitment and activation of cytotoxic lymphocytes, immunostimulatory myeloid cells, their synergy or non-immunological mechanisms. Analogous immunological considerations may not apply to treatment-naïve RMA, in which intratumoral CD54+ microvessels and immune cells were consistently sparse and in which the occurrence of even a few CD54+ microvessels was associated with a significantly poorer prognosis than their complete absence. The opposite prognostic association of CD54+ microvessel density in RME and RMA is a new difference among many others between RME and RMA and a new example of the paradigm that the prognostic impact of intratumoral microvessel density depends on tumor type^54–59^.

The current findings migh have therapeutic implications: (1) the consistent lack of PD-L1 on tumor cells and tumor infiltrating immune cells, and the paucity of PD1+ cells in the TME of all our RMS cases (n = 39) (in agreement with previous studies^33,60^), makes the random targeting of this immune checkpoint unlikely to be successful, while specific targeting may eventually be effective in the small, previously reported RMS subset with a PD-L1^high^ immunophenotype^45,61^. (2) Novel immunotherapeutic strategies aim to target the immunosuppressive and tumor-promoting function of tumor-infiltrating myeloid cells by blocking the recruitment of monocytes or other precursors^62^. Whether such a strategy can be beneficial in RMS is an open question in light of our finding that higher numbers of macrophages in the TME were associated with better survival, at least in RME patients. By contrast, the latter findings may be a rational for strategies that try to activate the phagocytic capacity of intratumoral macrophages^53^ or skew their polarization towards an immunostimulatory function^62^. (3) Taking the overall paucity of intratumoral CD3+ in RMS into account, it appears likely that the adoptive transfer of RMS-directed cytotoxic lymphocytes alone may be insufficient to eradicate established RMS. Indeed, we previously showed that human RMS xenografts are only transiently susceptible to RMS-specific chimeric T cells^14^. Therefore, combination strategies that improve the recruitment of lymphoid cells (including chimeric effector cells) to the TME of RMS and prevent their inactivation there may be necessary to improve cell based immunotherapies. To rationally design such complementary interventions, be they directed towards ‘anergic endothelial cells”^47^ or intratumoral myeloid cells will require a more in depth analysis of intratumoral as well as peritumoral microvessels and a more comprehensive characterization - beyond the simplistic CD68+ and CD163+ dichotomy - of the many different myeloid cell subsets that can now be distinguished by multiparameter analysis^48^.

## Material and Methods

### Biopsies

Paraffin embedded RMS biopsies from 39 patients, 20 diganosed with RMA and 19 with RME, were obtained from the Pediatric Tumor Registry, Kiel, Germany. All analyzed RMA tumors were PAX3-FOXO1 positive. Histopathology of all cases was centrally review by Professor Ivo Leuschner (Pediatric Tumor Registry, Kiel, Germany). All patients were treated according to CWS protocols. Written informed consent according to the Declaration of Helsinki was obtained from all patients or their legal guardians, depending on the patients’ age. All studies were approved by the appropriate ethics and review committees (approval number 158/2009/b02; University of Tübingen, Tübingen, Germany; April 2, 2009) and 2012-257N-MA (University of Heidelberg, University Medical Centre Mannheim, Mannheim; April 12, 2012)). Composition of the available dataset is summarized in Supplemental Table S4 and Fig. S4.

A set of human solid tumors for comparision of the tumor microenvironment in RMS and other tumor types were recently published by Kather *et al*.^23^.

### Immunohistochemistry

Immunohistochemistry staining was done on 0.5–1 µm slides of paraffin embedded RMS biopsies by using routine immune peroxidase techniques. For quality control reasons, tonsils were included as positive controls. Pretreatment of the sections is depending on the used antibody. Antibodies, pretreatment conditions and antibody dilutions are listed in Supplemental Table S5. Immunohistochemistry was performed as described^63^ using the chemicals and reagents listed below: antigen retrieval in Novocastra antigen retrieval solution ph6 or pH 9.0 (Leica, Wetzlar, Germany); blocking of endogenous peroxidase (DAKO blocking solution, DAKO, Hamburg, Germany); detection of bound antibodies by the immunoperoxidase/DAB-based DAKO REAL detection system (DAKO).

### Manual image analysis

All 39 paraffin embedded RMS tissues were assessed for the infiltration with T-lymphocytes by using CD3 and CD8 staining, tumor-associated monocytes/macrophages by using CD68, CD163 and CD11b or B cells by using CD20 staining. Regulatory T cells (Tregs) were analyzed by using FOXP3 staining. For manual, semi-quantitative cell enumeration, an expert pathologist (A.M.) reviewed all slides independently of the original pathology report and estimated each immune cell infiltrate as the percentage of a given immune cell subset (e.g. CD3+ cells) per one hundred tumor cells. All analyses were performed in a blinded way with respect to clinical outcome. The validity of this approach was ensured by correlating the percentages based on manual enumeration with the results of computer-based image analysis (see below). Immune cells were also subdivided in terms of their localization as “intra-tumoral” (i.e. in between tumor cells), “septal” (i.e. inside stromal septa that typically encircle tumor cell nodules mainly in RMA or peripheral (i.e. at the invasion front). In addition, follicular arrangement of lymphoid cells was assessed.

### Computer-based image analysis

We used a semi-automatic image processing pipeline to quantitatively assess the immune infiltrate. First, all tissue slides were digitized using a Aperio Digital Slide Scanner at 20x magnification (0.5 µm/pixel). Then a board certified pathologist (C.H.) manually delineated the tumor region in the digital slides. Next, we used QuPath v0.1.2^64^ to automatically detect all positively stained cells as described before^19^. The thresholds for cell detection were manually optimized for each type of staining and the same settings were used for all cases. For all further analyses, we used the mean cell density (cells per mm²) in the tumor tissue. For vessel detection a previously developed morphometric algorithm was used, that could safely recognized and distinguished endothelial cells from other CD31 und CD34 positive stromal cells^65^. Since CD34 expression is not restricted to endothelial cells, CD31 was applied as second endothelial marker compared to the CD34 pattern (Supplemental Fig. S3).

### Statistics

Logistic regression analysis and survival analysis were done using SAS statistical software, release 9.4 (SAS Inistute Inc., Cary, North Carolina, USA). Non-parametric tests were used to compare groups; the Mann-Whitney test to compare two and the Kruskal-Wallis-Test to compare more than two groups. For survival analysis a logrank test (Mantel-Cox test) was used. Graphics were done by using the GraphPad Prism Software tool. Significant results were defined as p < 0.05; trends with p < 0.1.

## Supplementary information

Supplemental Figures and Tables

## Data Availability

All data generated or analysed during this study are included in this published article (and its supplementary information files).
