# Supplementary material for: CD163+ immune cell infiltrates and presence of CD54+ microvessels are prognostic markers for patients with embryonal rhabdomyosarcoma
Source: Sci Rep. 2019 Jun 25;9:9211. doi: 10.1038/s41598-019-45551-y (PMC6592899; doi:10.1038/s41598-019-45551-y)
Supplement: Supplementary file 1 — Supplemental Figures and Tables [file 41598_2019_45551_MOESM1_ESM.pdf]

Supplemental figures

CD163+ immune cell infiltrates and presence of CD54+ microvessels are prognostic markers for patients with embryonal rhabdomyosarcoma

Jakob Nikolas Kather [1,2], Christian Hörner [3], Cleo-Aron Weis [3], Thiha Aung [4], Christian Vokuhl [5], Christel Weiss [6], Monika Scheer [7], Alexander Marx [3] and Katja Simon-Keller* [3]

Affiliation:
1: Applied Tumor Immunity, German Cancer Research Center, Heidelberg, Germany
2: Internal Medicine III, University Hospital RWTH Aachen, Aachen, Germany
3: Institute of Pathology, University Medical Center Mannheim, Mannheim, Germany
4: Center of Plastic-, Hand- and Reconstructive Surgery, University of Regensburg, Regensburg, Germany
5: Institute of Pathology, Paidopathology, University Medical Center Kiel, Kiel, Germany
6: Department of Medical Statistics and Biomathematics, University Medical Centre Mannheim, Mannheim, Germany
7: Pediatrics 5 (Oncology, Hematology, Immunology), Olgahospital, Klinikum Stuttgart, Stuttgart, Germany
* corresponding author: katja.simon-keller@medma.uni-heidelberg.de

S1

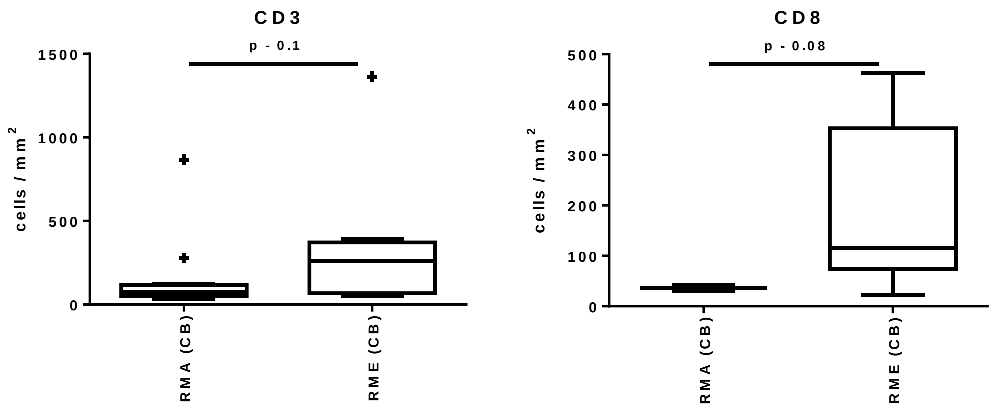

Supplemental Figure S1: Box and Whisker Blot for the quantification of infiltrating CD3 and CD8 immune cells in the tumor microenvironment of RME and RMA tumors after excluding low numbers (less than 20 cells/mm2) from the calculation.

S2

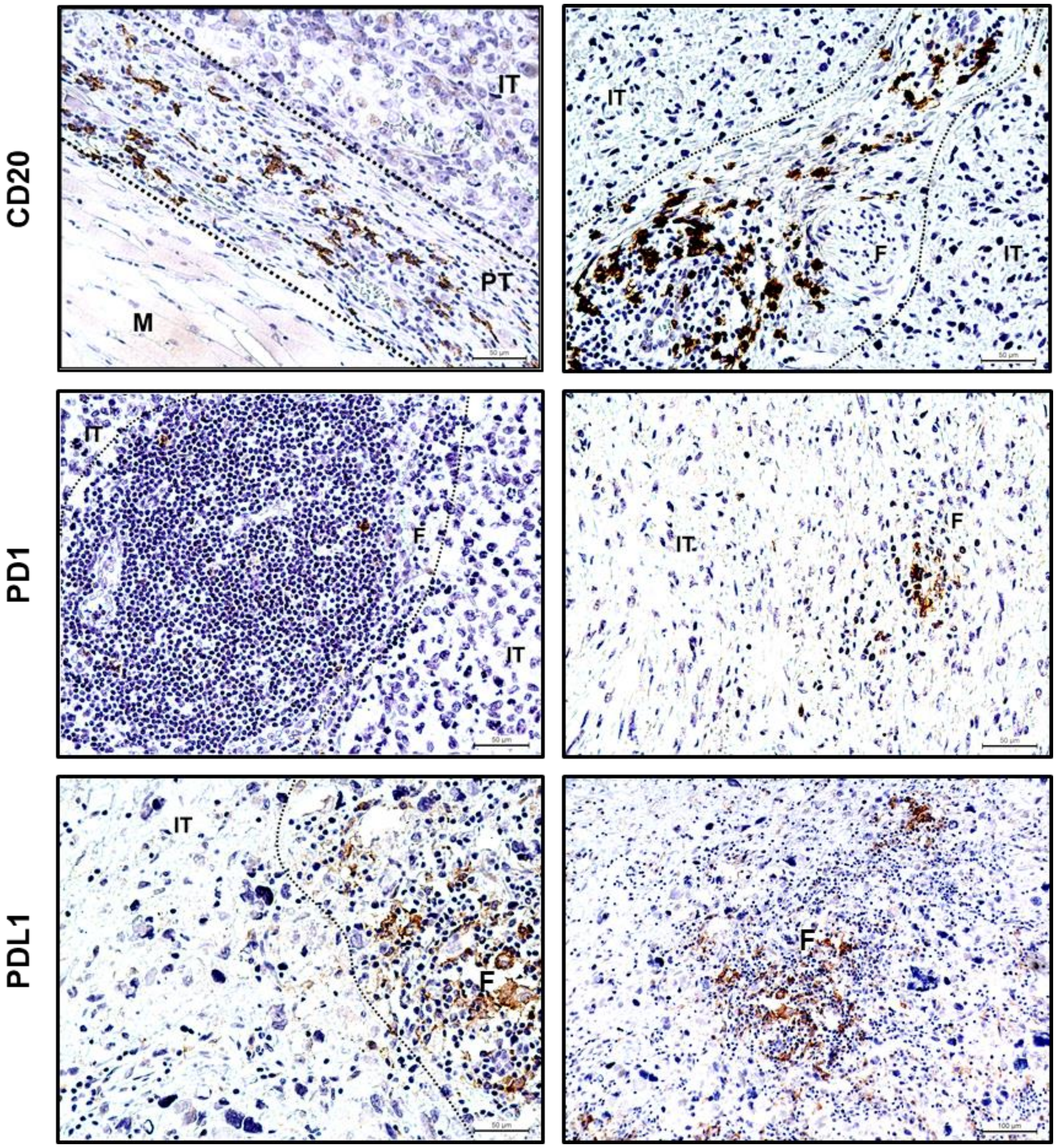

Supplemental Figure S2: shows staining of CD20, PD1 and PDL1 in peritumorous region and peritumoral lymphoid follicles; F- follicles IT – intratumoral, PT – peritumoral, M – skeletal muscle; magnification – 200x

S3

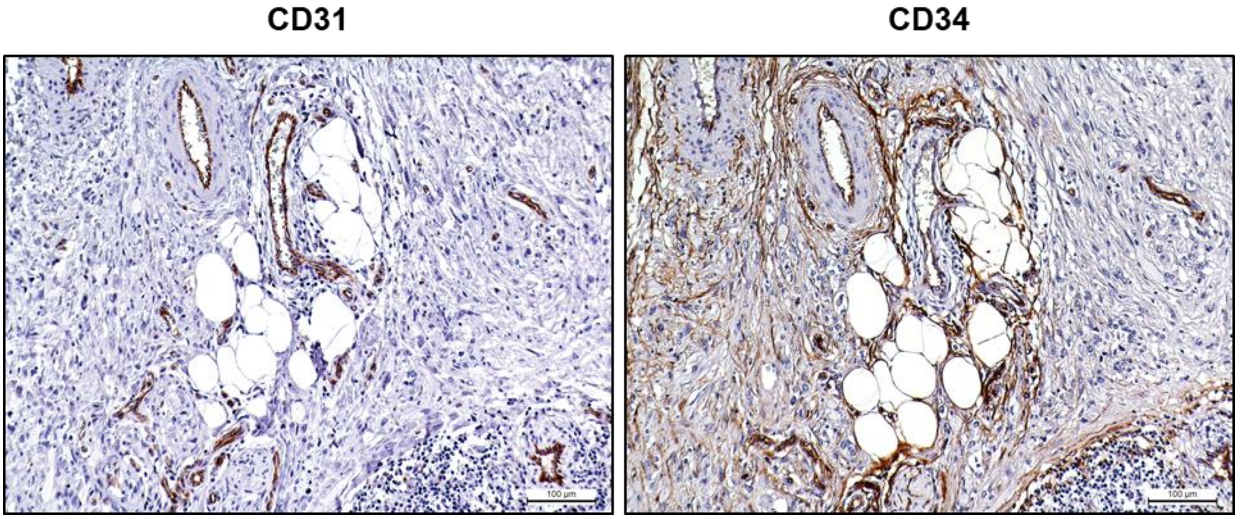

Supplemental Figure S3: shows a staining of CD31 and CD34 in a representative RMS tumor; magnification – 200x

S4

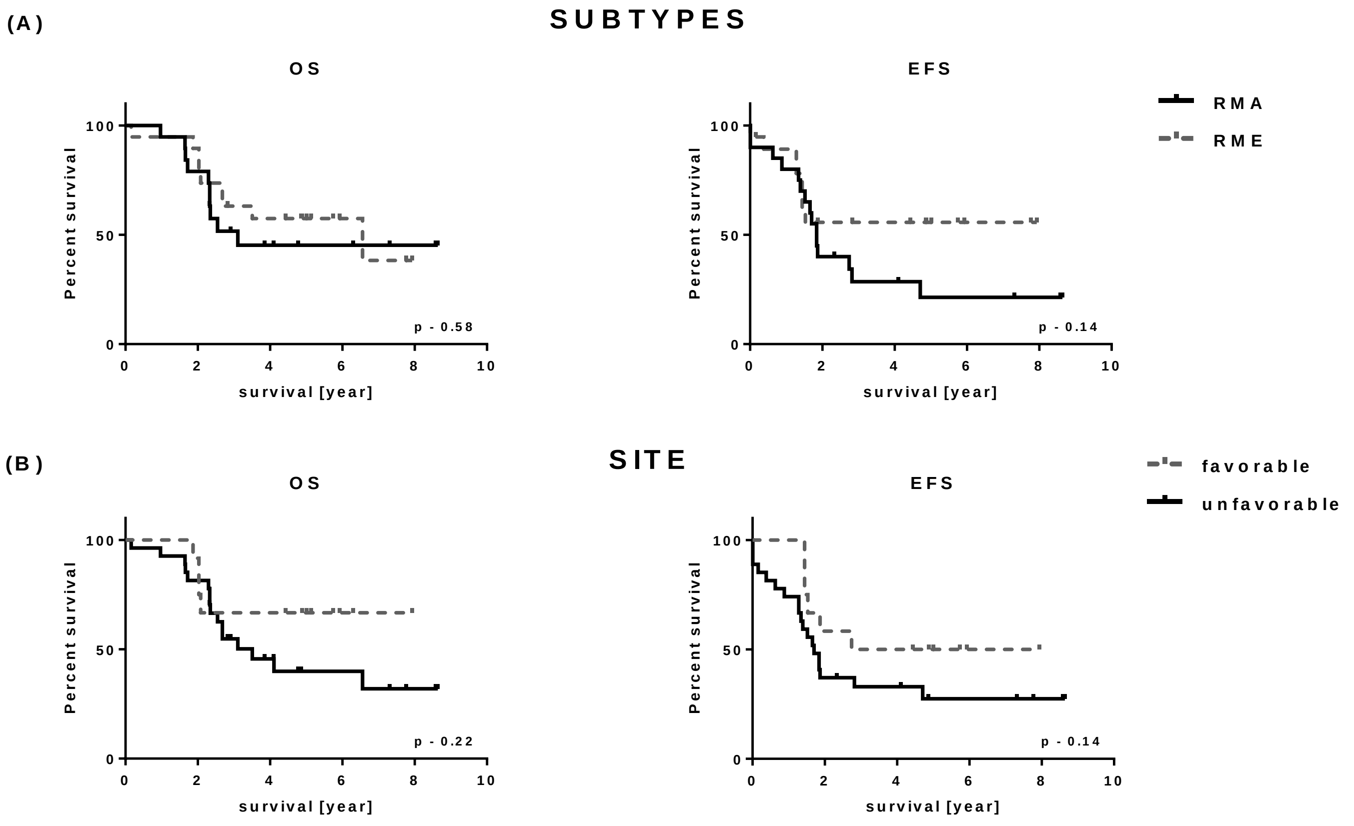

Supplemental Figure S4: (A) Comparision of OS and EFS of RMA and RME patients. (B) Comparision of OS and EFS of patients with more favorable and unfavorable tumor sites;
*favorable = Orbit/eye lid, head and neck (excluding parameningeal), genito-urinary (not bladder or prostate);
*unfavorable = Bladder, prostate, extremity, parameningeal, other (trunk, retroperitoneal).
OS – overall survival; EFS – event free survival

S5

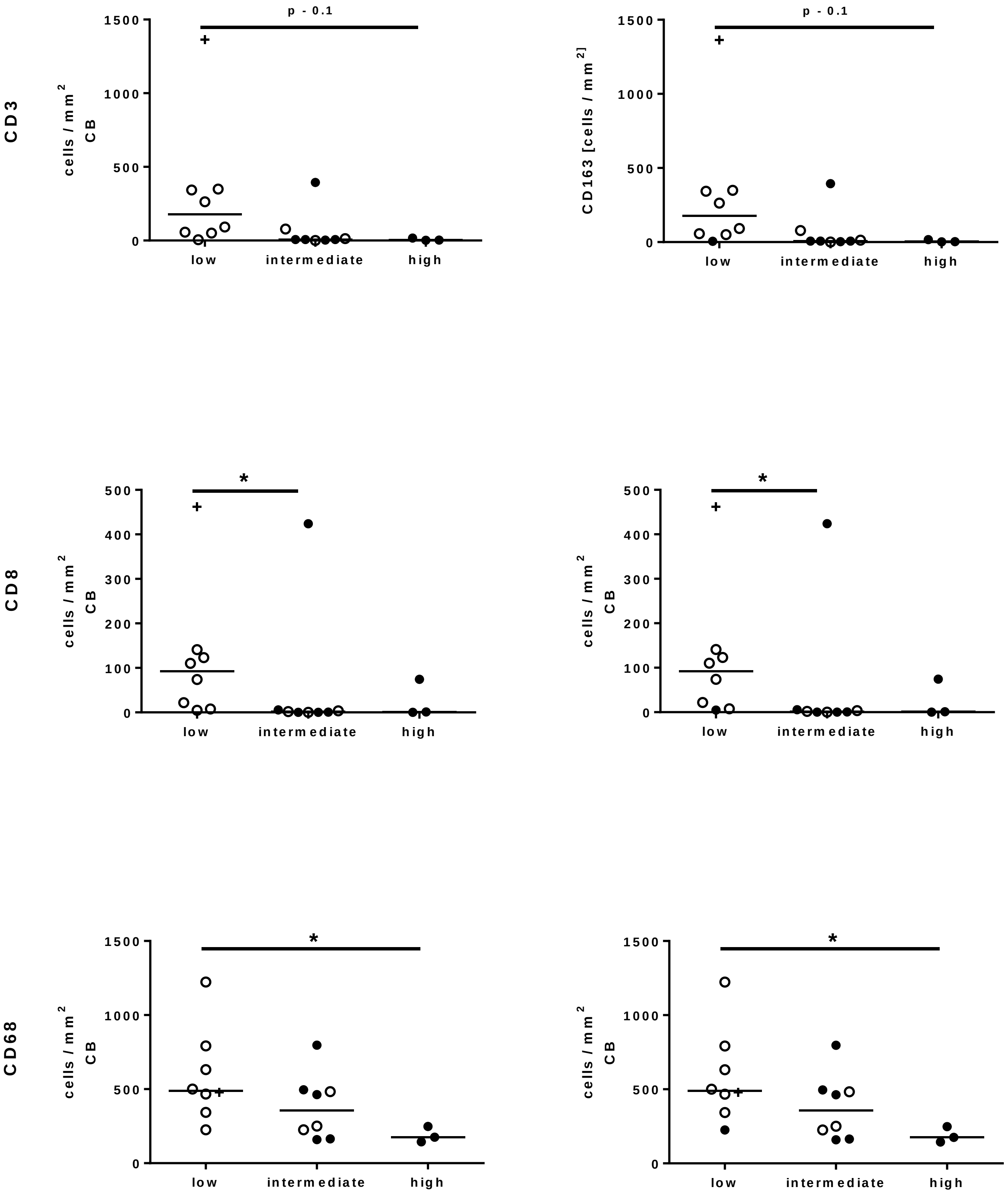

Supplemental Figure S5: shows the number of CD163 positive cells in the tumor microenvironment of low, intermediate and high risk RME patients. Due to a strong skrew distribution the median is shown. One patient (shown as cross) in the group “low” risk shows an unusually high density of immune cells and was not considered in the present statistical analysis (see also Supplemental Figure S5). Open circles represent patients who (left panel) did not die or (right panel) had no detectable event during the observation period. * p<0.05, ** 0.05 > p > 0.01, *** 0.01 > p > 0.005, **** p < 0.005.

Supplemental Tables

Supplemental Table S1: correlation coefficients are given (Pearson); significance are indicated as the following
* - p<0.05; ** 0.05>p>0.01; *** 0.01>p>0.005; **** 0.005>p>0.001

	RMS all	RMA	RME
CD3/CD8 association	0.857 ****	0.306	0.866****
CD68/CD163 association	0.512 **	0.474*	0.566*
CD3/CD68 association	0.402 *	0.636 ***	0.298
CD3/CD163 association	0.442 **	0.600 **	0.325

Supplemental Table S2: correlation coefficients are given (Pearson); significance are indicated as the following
* - p<0.05; ** 0.05>p>0.01; *** 0.01>p>0.005; **** 0.005>p>0.001

	RMS all	RMA	RME
CD34/CD3 association	0.287	0.002	0.324
CD34/CD8 association	0.693****	0.569*	0.681**
CD34/CD68 association	0.360*	0.415	0.303
CD34/CD163 association	0.159	0.146	0.157

Supplemental Table S3: Log Rank Test (p-values are given)

		RMS all	RMA	RME
$CD54^{HIGH}$ / $CD54^{LOW}$	OS	0.18	-	0.20
	EFS	0.17	-	0.05
CD54 (0) / CD54 (1)	OS	-	0.05	-
	EFS	-	0.02	-

Supplemental Table S4: statistical distribution of RMS subtypes regarding age, sex and clinical attributes

	RMS all	RMA	RME
	**39**	**20**	**19**
**SEX**			
♀	16	10	6
♂	23	10	13
**AGE**	7.7 (± 6.9)	8.25 (± 8.7)	7.1 (± 4.6)
**SITE**			
favorable*	12	1	11
unfavorable*	27	19	8
**TUMOR SIZE**			
< 5 cm	14	6	7
> 5 cm	25	14	12
**METASTASIS**			
no	30	15	15
yes	9	5	4
**LYMPHE NODE**			
infiltration	12	6	6
no infiltration	27	14	13
**DEATH**			
no	20	10	10
yes	19	10	9
**EVENT**			
no	14	5	11
yes	25	15	8

*favorable = Orbit/eye lid, head and neck (excluding parameningeal), genito-urinary (not bladder or prostate);
*unfavorable = Bladder, prostate, extremity, parameningeal, other (trunk, retroperitoneal).

Supplemental Table S5: primary antibodies used in the present work

ANTIBODY	CLONE	TREATMENT	DILUTION	COMPANY
CD3	SP7	EDTA	1/200	Thermo
CD8	4B11	EDTA	1/100	Leica
CD11b	EP1347Y	EDTA	1/25	abcam
CD20	L26	EDTA	1/500	Agilent
CD54	ICAM-1	EDTA	1/25	CellSignaling
CD68	PG M1	EDTA	1/300	Agilent
CD163	10D6	EDTA	1/50	Leica
DESMIN	polyclonal	pH6	1/2000	Thermo Fisher
FOXP3	polyclonal	pH6	1/40	DCS
PD-1	NAT105	pH6	1/50	abcam
PD-L1	E1L3N	EDTA	1/40	CellSignaling

Supplemental Table S6: comparison of tumor infiltrating lymphocytes in different tumor entities (literature). Median and (if available) mean numbers of tumor infiltrating cells are given.

RMS
(our own study)

	CD3	CD8	CD68	CD163
**MEDIAN**	47,88	2,17	270,37	259,95
**RMA**	50,03	2,03	235,97	119,37
**RME**	16,72	5,69	463,09	439,84
**MEAN**	128,54	43,80	363,34	349,79
**RMA**	97,80	9,10	291,49	303,07
**RME**	160,90	76,68	435,19	396,52

Neuroblastoma
(Mina, M. et al.; Oncoimmunology; 2015)

	CD3	CD8	CD68	CD163
**MEDIAN**	8,2*	3*		
**MEDIAN**	14,4**	9,5**		

*Nests **Septa

Medulloblastoma
(Vermeulen J.F. et al.; Oncoimmunology 2018)

	CD3	CD8	CD68	CD163
**MEDIAN**	23,5			

Synovial sarcoma
(Oike et al.; Cancer science; 2018), adult and childhood

	CD3	CD8	CD68	CD163
**MEDIAN**		188,3		444

Bladder carcinoma
(Kather J.N. et al.; eLIFE 2018)

	CD3	CD8	CD68	CD163
**MEDIAN**	184,83	181,20	336,63	495,52
**MEAN**	361,06	620,40	507,65	701,09

Colorectal adenocarcinoma
(Kather J.N. et al.; eLIFE 2018)

	CD3	CD8	CD68	CD163
**MEDIAN**	105,40	87,94	179,21	160,04
**MEAN**	249,84	156,43	232,57	223,17

Esophageal squamous carcinoma
(Kather J.N. et al.; eLIFE 2018)

	CD3	CD8	CD68	CD163
**MEDIAN**	49,99	153,20	194,63	252,34
**MEAN**	106,29	226,51	271,11	393,18

Head and neck squamous cell carcinoma
(Kather J.N. et al.; eLIFE 2018)

	CD3	CD8	CD68	CD163
**MEDIAN**	278,25	205,82	333,52	456,80
**MEAN**	622,40	378,78	420,23	505,52

Lung adenocarcinoma
(Kather J.N. et al.; eLIFE 2018)

	CD3	CD8	CD68	CD163
**MEDIAN**	486,97	331,59	762,76	572,31
**MEAN**	613,57	459,76	977,61	648,24

Lung squamous cell carcinoma
(Kather J.N. et al.; eLIFE 2018)

	CD3	CD8	CD68	CD163
**MEDIAN**	353,39	320,46	272,99	535,46
**MEAN**	388,36	381,51	282,29	561,53

Melanoma
(Kather J.N. et al.; eLIFE 2018)

	CD3	CD8	CD68	CD163
**MEDIAN**	227,95	479,13	400,72	722,21
**MEAN**	916,27	561,65	610,84	780,70

Ovarian cancer
(Kather J.N. et al.; eLIFE 2018)

	CD3	CD8	CD68	CD163
**MEDIAN**	11,82	40,29	25,50	122,87
**MEAN**	26,90	153,08	23,57	174,68

Gastric cancer
(Kather J.N. et al.; eLIFE 2018)

	CD3	CD8	CD68	CD163
**MEDIAN**	241,21	367,72	200,16	432,42
**MEAN**	432,71	751,19	415,45	548,78
